# Microfluidic Chip for Quantitatively Assessing Hemorheological Parameters

**DOI:** 10.3390/mi16050567

**Published:** 2025-05-08

**Authors:** Yang Jun Kang

**Affiliations:** Department of Mechanical Engineering, Chosun University, 10, Chosundae 1-gil, Dong-gu, Gwangju 61452, Republic of Korea; yjkang2011@chosun.ac.kr; Tel.: +82-62-230-7052; Fax: +82-62-230-7055

**Keywords:** microrheology, microfluidic chip, blood viscosity, RBC aggregation, RBC sedimentation rate

## Abstract

The biomechanical properties of blood are regarded as promising biomarkers for monitoring early-stage abnormalities and disease progression. To detect any changes in blood, it is necessary to measure as many rheological properties as possible. Herein, a novel method is proposed for measuring multiple rheological properties of blood using a microfluidic chip. The syringe pump turns off for 5 min to induce RBC (red blood cell) sedimentation in the driving syringe. RBC aggregation is determined by analyzing the time-lapse blood image intensity at stasis: *I*(*t*) = *I*_1_ exp (−*k*_1_ *t*) + *I*_2_ exp (−*k*_2_ *t*). RBC-rich blood and RBC-depleted blood are sequentially infused into the microfluidic chip. Based on blood pressure estimated with time-lapse blood velocity, blood viscosity is acquired with the Hagen–Poiseuille law. RBC sedimentation is quantified as RBC sedimentation distance (*X_esr_*) and erythrocyte sedimentation rate (ESR). The proposed method provides a consistent viscosity compared with previous methods. Two of the four variables (*I*_1_, *I*_2_) exhibited a strong correlation with the conventional RBC aggregation index (AI). The indices *X_esr_* and ESR showed consistent trends with respect to the blood medium and hematocrit. In conclusion, the proposed method is then regarded as effective for monitoring multiple rheological properties.

## 1. Introduction

The biomechanical properties of blood, including its viscosity and elasticity, are regarded as promising biomarkers for detecting early-stage abnormalities and monitoring disease progression [1,2,3,4,5,6,7]. They have a substantial influence on vascular resistance [8,9], oxygen delivery [10,11], and tissue perfusion [12,13]. According to clinical studies, several diseases, such as, hypertension [14], diabetes [15,16], and atherosclerosis [17], contribute to increased blood viscosity, red blood cell (RBC) aggregation, and high blood pressure. According to previous studies [15,18,19], the rheological properties of blood can be determined from plasma proteins and RBCs (i.e., hematocrit (Hct) and membrane viscoelasticity). Therefore, to detect any changes in the blood (i.e., plasma proteins or RBCs), it is necessary to measure as many rheological properties as possible using the same device.

Since the introduction of microfluidic chips in blood rheological studies, several methods have been devised for effective quantification of blood rheological properties [20]. As a fundamental property of blood, its viscosity (i.e., plasma, whole blood, or suspended blood) has been obtained by measuring the droplet length [21], color index in the droplet [22], microflow compartment [23], reversal flow in the bridge circuit [6], coflowing streams [24], blood-flow time [25,26], blood filling time [27,28], micropillar deflection [29], acoustic oscillation [30], light scattering [31], frequency shift under fluid mass [32], and capillary pressure in the microfluidic channel [33,34,35,36,37]. However, it is extremely difficult to induce RBC aggregation at higher flow rates when blood flows in the microfluidic channel. To investigate the contribution of the blood medium or RBC viscoelasticity to RBC aggregation, blood flow is stopped to induce RBC aggregation at low shear rates [18,38]. The RBC aggregation index (AI) is then acquired by analyzing syllectogram (light transmission or reflection) [39,40,41], microscopic image intensity [42], electric impedance [43,44,45], optical tweezer [46], and photoacoustic ultrasound [47]. After the blood is loaded into a vertically installed tube, RBCs begin to aggregate and form an interface layer between the plasma and RBCs. The interface tends to move downward over time along the gravitational direction. The conventional RBC sedimentation method requires a large amount of blood (mL) and a long waiting time (h). Most importantly, it is difficult to acquire a time-lapse interface because the interface in the tube is not clearly visible. To resolve the issues associated with conventional methods, several methods have been proposed for quantifying RBC sedimentation using microfluidic device [48,49,50,51,52]. Most of the current methods are suitable for extracting only a single blood parameter. Furthermore, it is difficult to directly investigate the effects of RBC aggregation and sedimentation on blood viscosity because Hct changes continuously over time. Previously, by maintaining the blood flow rate using two syringe pumps, blood viscosity has been determined by monitoring the interface between fluids under varying Hct conditions [53]. Additionally, under pulsatile flow rate controlled by the syringe pump, three rheological properties (i.e., viscoelasticity, RBC aggregation, and blood pressure) have been acquired with a microfluidic chip [54]. However, the previous study does not obtain quantitative information on blood viscosity by measuring blood pressure. Its mathematical representation is still insufficient for explaining RBC aggregation and sedimentation [55].

In this study, a novel method is proposed for measuring the contribution of RBC sedimentation in a driving syringe to blood microrheology using a microfluidic chip. A syringe pump was used to load blood into the microfluidic channel at a constant blood flow rate. The syringe pump was turned off to induce RBC sedimentation in the driving syringe. RBC aggregation was determined by analyzing time-lapse image intensity at stasis. Based on a simple kinetic model of RBC aggregation, four variables (*I*_1_, *I*_2_, *k*_1_, *k*_2_) for representing RBC aggregation were acquired by the best fitting time-lapse intensity as *I*(*t*) = *I*_1_ exp (−*k*_1_*t*) + *I*_2_ exp (−*k*_2_*t*). After a certain elapse of time, RBC sedimentation contributed to the formation of RBC-depleted and RBC-rich blood in the driving syringe. By turning on the syringe pump, the RBC-rich and RBC-depleted blood samples were sequentially loaded into the microfluidic chip. Blood pressure was then determined by analyzing the blood flow within the air damper. Time-lapse blood viscosity was calculated using the Hagen–Poiseuille law (pressure drop = fluidic resistance × flow rate). When RBC-depleted blood is loaded into a microfluidic channel, the blood viscosity or image intensity exhibits substantial changes over time. Thus, time-lapse blood velocity and image intensity were analyzed to obtain two parameters (i.e., *X_esr_*: RBC-free height in the driving syringe, ESR = *X_esr_*/overall blood delivery time) of RBC sedimentation in the driving syringe

Unlike previous methods, the proposed method can sequentially measure at least three vital parameters of blood (RBC aggregation, blood viscosity, and RBC sedimentation) in the same microfluidic chip. After RBC sedimentation occurs in the driving syringe, RBC-rich blood and RBC-depleted blood are sequentially loaded into the microfluidic device. By inspecting the time-lapse image intensity of the blood, it is possible to quantify RBC aggregation (at stasis) and RBC sedimentation in the driving syringe (blood flow). New variables are introduced: (*I*_1_, *I*_2_, *k*_1_, *k*_2_) for RBC aggregation and (*X_esr_*, ESR) for RBC sedimentation. The blood velocity in the compliance channel connected to an air damper is used to measure the pressure of the blood flow. Considering that the blood flow rate remains unchanged over time with the syringe pump, the blood velocity in the guiding channel of the air damper is calibrated with the blood velocity delivered by the syringe pump. Therefore, instead of using two syringe pumps, the blood viscosity can be obtained from the time-lapse blood pressure by supplying blood with a single syringe pump. Furthermore, because blood is loaded into a microfluidic channel after RBC sedimentation, RBC sedimentation (or RBC aggregation) can contribute to blood viscosity.

## 2. Materials and Methods

### 2.1. Experimental Setup for Micro Hemorheology with Microfluidic Chip

**Figure 1:**
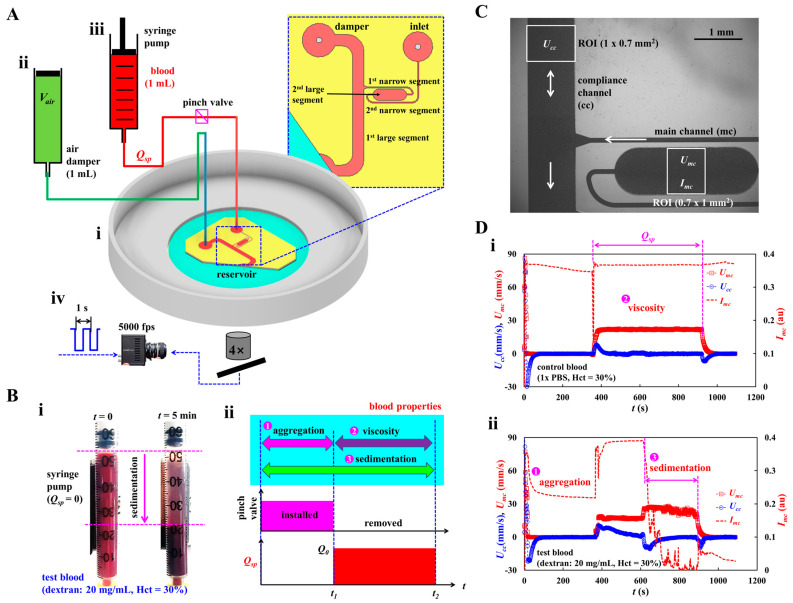
Novel method for acquiring hemorheological properties in microfluidic channel. (**A**) Schematic diagram of experimental setup, including, a microfluidic chip, single syringe pump, an air-damper, and microscopic image acquisition system. (**i**) A microfluidic chip was composed of inlet port, main channel (mc), compliance channel (cc), air-damper port, and reservoir. (**ii**) The air damper (cavity = 1 mL) as a pressure sensor. (**iii**) A syringe pump for loading blood sample into the microfluidic chip at the constant flow rate (*Q_sp_*). To stop blood flow inside the tubing, the tubing was obstructed with a pinch valve. (**iv**) Microscopic image acquisition system, such as an inverted microscope (4×, NA = 0.1), and a high-speed camera. Based on external triggering, two sequential microscopic images were obtained at an interval of 1 s. The high-speed camera set to 5000 frames per second. (**B**) RBC (red blood cell) sedimentation in the driving syringe and its impact quantification in terms of three properties (i.e., aggregation, viscosity, and sedimentation). (**i**) RBC sedimentation in the driving syringe. (**ii**) Quantification of three blood properties. First, by turning off syringe pump (*Q* = 0, *t* < *t*_1_) and obstructing the Tygon tubing with a pinch valve, RBC aggregation was obtained at stasis. Second, by removing the pinch valve and turning on the syringe pump (*Q* = *Q_sp_*, *t*_1_ < *t* < *t*_2_), blood viscosity as well as RBC sedimentation were acquired under continuous blood flow. (**C**) Quantification of image intensity (*I_mc_*) and blood velocity (*U_mc_*, *U_cc_*). The same size of ROI selected in each channel was adjusted to 0.7 mm^2^. (**D**) The impact of RBC sedimentation on the blood velocity and image intensity. (**i**) Temporal variations of *U_mc_*, *U_cc_*, and *I_mc_* for control blood. (**ii**) Temporal variations of *U_mc_*, *U_cc_*, and *I_mc_* for test blood.

To investigate multiple hemorheological properties, as shown in Figure 1A, the experimental setup consisted of a microfluidic chip, a single syringe pump for loading blood, an air damper for sensing pressure, and a microscopic image acquisition system.

Figure 1(Ai) illustrates a microfluidic chip composed of an inlet port, main channel (mc), compliance channel (cc), air-damper port, and reservoir. The main channel consisted of four segments connected in series: the first large segment (width [*w*] = 1000 μm, length [*l*] = 8685 μm) connected to the reservoir, the first narrow segment (width = 100 μm, length = 4929 μm), the second large segment (width = 1000 μm, length = 2995 μm), and the second narrow segment (width = 100 μm, length = 8916 μm) connected to the inlet port. Herein, blood viscosity was acquired in the first large channel. Additionally, RBC aggregation, and RBC sedimentation were measured in the second large channel.

A compliance channel (width = 1000 μm, length = 5795 μm) was connected to the end junction of the first large segment of the main channel. The channel depth was set to *h* = 50 μm. A four-inch silicon master mold was fabricated using photolithography and deep reactive-ion etching. A polydimethylsiloxane (PDMS; Sylgard 184, Dow Corning, Midland, MI, USA) block was replicated using soft lithography. The inlet and air-damper ports were created using a biopsy punch (outer diameter = 0.75 mm). The outlet port was cut partially with a razor blade and exposed to the reservoir without being connected to the tubing. An oxygen plasma system was used to bond the PDMS block to a glass-bottom dish of well size = 30 mm (Cellvis, Mountain View, CA, USA). One Tygon tubing (inner diameter = 250 μm, length = 300 mm) was connected to the inlet port. The other Tygon tubing (inner diameter = 700 μm, and length = 200 mm) was connected to the air-damper port. To avoid the nonspecific bonding of plasma proteins to the inner surface of the microfluidic channels, bovine serum albumin (BSA, 50 mg/mL) was loaded into the microfluidic channels through the inlet port. After 10 min, 1× phosphate-buffered saline (PBS) was loaded into the microfluidic channels by discharging BSA.

As shown in Figure 1(Aii), to secure the air cavity inside the disposable syringe (*V_air_* = 1 mL), an air damper was prepared by moving and fixing the plunger with a steel pin. The air damper measured the blood pressure in the microfluidic channel. The outlet of the air damper was connected to the other end of Tygon tubing fitted to the air-damper port. As shown in Figure 1(Aiii), the driving syringe was filled with blood (approximately 1 mL). The syringe outlet was connected to the other end of the Tygon tubing fitted to the inlet port. To discharge the air bubbles in the fluidic paths (i.e., syringe needle, Tygon tubing, and inlet port), blood was supplied to the microfluidic channels by pushing the plunger manually. It is necessary to ensure that no air bubbles are present in the microfluidic channels. The syringe was installed in a syringe pump (neMESYS, Cetoni GmbH, Korbußen, Germany) aligned along the direction of gravity. Before turning on the syringe pump, to stop the blood flow in the microfluidic channel, a pinch valve was installed to obstruct the blood flow in the Tygon tubing (i.e., blood flow stoppage). After removing the pinch valve, the flow rate of the syringe pump was set to a constant value (*Q* = *Q_sp_*).

To image blood flow in the microfluidic channels (i.e., main and compliance channels), as shown in Figure 1(Aiv), the microscopic image acquisition system comprised an inverted microscope (IX81, Olympus, Tokyo, Japan) and a high-speed camera (FASTCAM MINI, Photron, Tokyo, Japan). The microfluidic chip was placed on the moving stage of the microscope. The objective lens was set to 4× (NA = 0.1). The high-speed camera was set to 5000 frames per second (fps). To capture two sequential images at intervals of 1 s, a functional generator generated triggering signals for the camera (i.e., saw-wave profile, period = 1 s). All experiments were conducted at room temperature (25 °C).

### 2.2. Suggested Protocols for Quantifying Multiple Hemorheological Properties

As shown in Figure 1B, novel protocols were suggested to effectively measure the contribution of RBC sedimentation in the driving syringe to the hemorheological properties in the microfluidic channel. The rheological properties of blood were acquired in sequential order (i.e., RBC aggregation, blood viscosity, and RBC sedimentation) by analyzing the time-lapse image intensity and blood velocity.

The two snapshots in Figure 1(Bi) show RBC sedimentation in the driving syringe when the syringe pump is turned off. To briefly explain the proposed protocols, the test blood (Hct = 30%) was prepared by adding normal RBCs to a dextran solution (20 mg/mL). Test blood was loaded into a disposable syringe. A driving syringe was installed in a syringe pump. The syringe pump was stopped to induce RBC sedimentation in the driving syringe. After 5 min, RBC sedimentation divided the test blood into RBC-depleted and RBC-rich blood. When the flow rate of the syringe pump was set to *Q* = *Q_sp_*, the RBC-rich and RBC-depleted blood were sequentially loaded into the microfluidic chip. The viscosity of the RBC-rich blood reflects the effect of RBC sedimentation in the driving syringe (i.e., Hct variation). As shown in Figure 1(Bii), the novel protocols allowed us to obtain three types of blood properties, namely, RBC sedimentation, RBC aggregation, blood viscosity, in sequential order. First, to induce RBC sedimentation in the driving syringe, the syringe pump was turned off for a specific duration (*t*_1_ = 5 min). Herein, the microfluidic channels were already filled with the test blood. Although the syringe pump is turned off, the gravitational force continuously generates a small amount of blood flow in the driving syringe. Continuous blood flow makes it difficult to obtain consistency in RBC aggregation. Therefore, it is necessary to completely stop the blood flow. To stop the blood flow, the Tygon tubing connected to the inlet port was obstructed by installing a pinch valve. RBC aggregation in the test blood was acquired by analyzing the time-lapse image intensity. Second, the pinch valve was removed to load the test blood into the microfluidic channels. The flow rate was set to *Q* = *Q_sp_* from *t* = *t*_1_ to *t* = *t*_2_. The flow rate and blood pressure were calculated over time by probing the blood velocities in the main and compliance channels. Blood viscosity was obtained by substituting both the flow rate and blood pressure into the Hagen–Poiseuille equation. At last, RBC sedimentation also occurred continuously during blood loading period (i.e., *t*_2_-*t*_1_ = *V_blood_*/*Q_sp_*). That is, RBC sedimentation was monitored during overall period of *t*_2_ (i.e., turn-off syringe pump: *t*_1_, and turn-on syringe pump: *t*_2_-*t*_1_). When RBC-depleted blood began to flow in the main channel, it was possible to calculate RBC sedimentation in the driving syringe. The blood velocity and blood image intensity were then adopted to measure RBC sedimentation in the driving syringe.

### 2.3. Blood Velocity and Blood Image Intensity in Microfluidic Chip

To determine the blood flow rates in the main and compliance channels, the blood velocity was measured in both channels. In addition, to measure RBC aggregation and sedimentation, the blood image intensity was measured in the main channel.

As shown in Figure 1C, to obtain the average blood velocity, a specific ROI (0.7 mm^2^) was selected within the main channel (mc) and compliance channel (cc). Blood velocity fields were acquired using an open-source micro-PIV program [56]. The corresponding average velocity of each channel was calculated and denoted as *U_mc_* (main channel) and *U_cc_* (compliance channel). The flow rate of the syringe pump set to a constant value (*Q* = *Q_sp_*) and the same blood was flowed into both channels. The flow rate in the compliance channel was estimated as *Q_cc_* = (*U_cc_*/<*U_mc_*>) × *Q_sp_*, where <*U_mc_*> is the overall average velocity of *U_mc_* during blood delivery.

**Figure A1:**
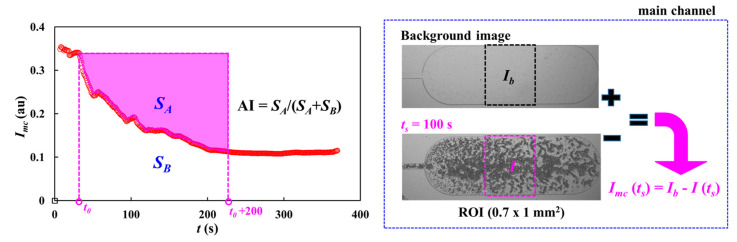
Calculation procedure of image intensity of each blood image captured at a specific time and syllectogram for quantifying RBC aggregation index (AI). The left-side panel showed temporal variations in blood image intensity. The right-side panel showed calculation procedure of image intensity: *I_mc_*(*t_s_*) = *I_b_* – *I*(*t_s_*). Herein, *I_b_* and *I* were calculated by analyzing background image and an image captured at *t* = *t_s_*, respectively.

To obtain time-lapse image intensity of blood in the main channel, a specific ROI (0.7 mm^2^) was selected within the second large segment of the main channel. The image intensity within the specific ROI was acquired using MATLAB 2024 (MathWorks; Natick, MA, USA). The right-side panel of Figure A1 (Appendix A) showed image intensity calculation. To obtain contribution of RBCs within the specific ROI, it was necessary to subtract image intensity of each image obtained at a specific time (*t* = *t_s_*) from one of background image. That is, based on the specific ROI, averaged image intensity of each image was obtained as *I_b_* (background image) and *I* (microscopic image at *t* = *t_s_*), respectively. Image intensity was then calculated as *I_mc_* (*t_s_*) = *I_b_* − *I*(*t_s_*) at a specific time.

Figure 1D shows the temporal variations in *U_mc_*, *U_cc_*, and *I_mc_* for the control and test blood. Blood samples (Hct = 30%) were prepared by adding normal RBCs to 1× PBS (control blood) and dextran solution (20 mg/mL) (test blood). Figure 1(Di,ii) show the temporal variations in *U_mc_*, *U_cc_*, and *I_mc_* for the control and test blood, respectively. When the syringe pump was turned off (*Q* = 0), RBC aggregation significantly decreased the image intensity over time. When the syringe pump was turned on (*Q* = *Q_sp_*), *U_mc_* reached the target value and remained unchanged until the pump was turned off. *U_cc_* increased substantially within a short period, then it gradually decreased and stopped changing. After confirming that *U_cc_* stopped and *U_mc_* remained unchanged, the blood viscosity was obtained using the Hagen–Poiseuille equation [57]. In addition, when the RBC-depleted blood entered the microfluidic channel, *U_mc_* increased substantially because Hct decreased significantly. *U_cc_* flowed downstream (i.e., negative sign). Furthermore, *I_mc_* decreased substantially over time. Both the RBC-rich and RBC-depleted blood exhibited substantial differences in the velocity profile and image intensity over time, as evident from the time-lapse measurements.

### 2.4. Previous Protocols for Acquiring Blood Viscosity and RBC Aggregation

A previously developed method (i.e., the coflowing method [24]) was used to compare the blood viscosity obtained using the method suggested in this study. In addition, the conventional RBC aggregation index (AI) [39] was used to compare the four parameters obtained using the proposed protocols.

**Figure A2:**
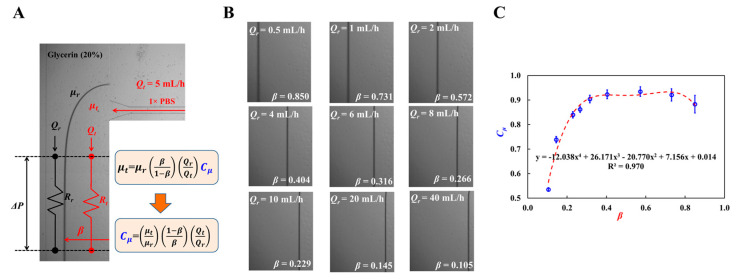
Coflowing method as previous method for measuring fluid viscosity. (**A**) Fluidic circuit representation using fluidic resistance (*R_r_*, *R_t_*) and flow rate (*Q_r_*, *Q_t_*). (**B**) Variation in β with respect to *Q_r_*. (**C**) Variations of *C_μ_* with respect to *β*. Polynomial expression of *C_μ_* was acquired as *C_μ_* = −12.038 *β*^4^ + 26.171 *β*^3^ − 20.770 *β*^2^ + 77.156 *β* + 0.014 (R^2^ = 0.970).

The coflowing-stream method was employed to measure the fluid viscosity in the microfluidic channel. The microfluidic channel proposed in this study was reused to detect the interface between the two streams. In this study, the damper port was designated as the inlet port for the reference fluid. Two syringe pumps were used to load the reference and test fluids into the microfluidic channel. As shown in Figure A2A (Appendix A), a fluidic circuit model was constructed in terms of fluidic resistance (*R_r_*, *R_t_*) and flow rate (*Q_r_*, *Q_t_*). 1× PBS and glycerin (20%) were used as the test and reference fluids, respectively. Interface between two streams was defined as *β* = stream width of test fluid/channel width. For convenience, the flow rate of the test fluid was maintained constant (*Q_t_* = 5 mL/h). Because both parallel streams had the same pressure drop (i.e., Δ*P_t_* = Δ*P_r_*), the viscosity formula of test fluid was derived as $\mu_{t}=\mu_{r}\left(\frac{\beta }{1-\beta }\right)\left(\frac{Q_{r}}{Q_{t}}\right)C_{\mu }$, where *C_μ_* is the correction factor to compensate for the modeling error induced owing to model simplicity for real parallel streams. Using viscosities *μ_r_* = 2 cP (reference fluid) and *μ_t_* = 1 cP (test fluid), *C_μ_* was estimated with respect to interface *β* as $C_{\mu }=\left(\frac{\mu_{t}}{\mu_{r}}\right)\left(\frac{1-\beta }{\beta }\right)\left(\frac{Q_{t}}{Q_{r}}\right)$. As shown in Figure A2B (Appendix A), variations in *β* were measured with respect to flow rate *Q_r_* of the reference fluid: *β* = 0.850 (*Q_r_* = 0.5 mL/h), *β* = 0.731 (*Q_r_* = 1 mL/h), *β* = 0.572 (*Q_r_* = 2 mL/h), *β* = 0.404 (*Q_r_* = 4 mL/h), *β* = 0.316 (*Q_r_* = 6 mL/h), *β* = 0.266 (*Q_r_* = 8 mL/h), *β* = 0.229 (*Q_r_* = 10 mL/h), *β* = 0.145 (*Q_r_* = 20 mL/h), and *β* = 0.105 (*Q_r_* = 40 mL/h). Figure A2C (Appendix A) shows the variations in *C_μ_* with respect to *β*. Using linear regression analysis, the correction factor was obtained as *C_μ_* = −12.038 *β*^4^ + 26.171 *β*^3^ − 20.770 *β*^2^ + 77.156 *β* + 0.014 (R^2^ = 0.970). The test fluid viscosity was then measured using the interface and flow rate of the reference fluid with the viscosity formula.

To quantify RBC aggregation in the test blood, the conventional RBC aggregation index (AI) was acquired by analyzing the time-lapse image intensity. As shown in the left-side panel of Figure A1 (Appendix A), after turning off the syringe pump, time-lapse blood image intensity (*I_mc_*) was acquired over time. Here, integration time set to 200 s from the specific time when the *I_mc_* (*t*) began to decrease (i.e., from *t*_0_ to *t*_0_ + 200 s). Two parameters (*S_A_* and *S_B_*) [33] were then obtained by analyzing the syllectogram. *S_A_* denoted the contribution of RBC aggregation, and tended to increase depending on the degree of RBC aggregation. The RBC aggregation index (AI) was expressed as a dimensionless parameter using AI = *S_A_*/(*S_A_* + *S_B_*).

### 2.5. Test-Blood Preparation for Performance Evaluation

The study was conducted in accordance with the principles of the Declaration of Helsinki. The Chosun University Ethics Committee approved this study (reference code: 2-1041055-AB-N-01-2021-80). Concentrated normal RBCs were purchased from the Gwangju–Chonnam Blood Bank (Gwangju, Republic of Korea) and stored in a refrigerator before conducting the experiments. For the washing protocol, concentrated normal RBCs were diluted 50% with 1× PBS (i.e., volume of RBCs: volume of 1× PBS = 1:1). The suspended blood was dropped on the cell strainer (mesh size = 50 μm) and filtered using gravity. The filtered blood was collected in a centrifuge tube. After centrifugation (4000 rpm for 10 min), pure normal RBCs were collected by removing the liquid and buffer layers. This procedure was repeated twice.

Control blood was prepared by adding normal RBCs to the medium (1× PBS, dextran solution). The Hct of the control blood was adjusted between 20 and 60%. Dextran solution was used as the blood medium to stimulate RBC aggregation in the test blood. The specific concentration of the dextran solution, *C_dex_*, was adjusted between 5 and 20 mg/mL by diluting dextran powder (*Leuconostoc* spp., M.W. = 450–650 kDa, Sigma-Aldrich, Saint Lousis, MO, USA) in 1× PBS. The test blood was prepared by adding normal RBCs to the dextran solution. To adjust the deformability of normal RBCs, three concentrations of diluted glutaraldehyde solution (*C_ga_* = 1, 2, and 4 μL/mL) were prepared by adding glutaraldehyde solution (Grade II, 25% in H_2_O, Sigma-Aldrich, USA) to 1× PBS. Normal RBCs were then added to the three diluted glutaraldehyde solutions. The suspended blood was then stirred for 30 min. The hardened RBCs were collected by removing the fluid after centrifugation. The test blood was prepared by adding the hardened RBCs to a dextran solution (20 mg/mL).

## 3. Results and Discussion

### 3.1. Quantification Procedures of RBC Aggregation, RBC Sedimentation, and Viscosity

Herein, overall quantification procedures were explained. That is, three types of blood rheological properties (RBC aggregation, RBC sedimentation, and blood viscosity) were obtained sequentially by analyzing time-lapse blood velocity and image intensity. First, by stopping the blood flow in the microfluidic channel, RBC aggregation was acquired by analyzing the time-lapse image intensity of blood in the microfluidic chip. Second, by loading blood into a microfluidic chip at a constant flow rate, the blood viscosity was obtained by analyzing the blood velocity in the compliance channel. The volume of RBC-depleted blood was evaluated by analyzing the time-lapse blood velocity and image intensity. The RBC sedimentation rate (ESR) was estimated from the volume of RBC-depleted blood during the entire blood delivery period.

**Figure 2:**
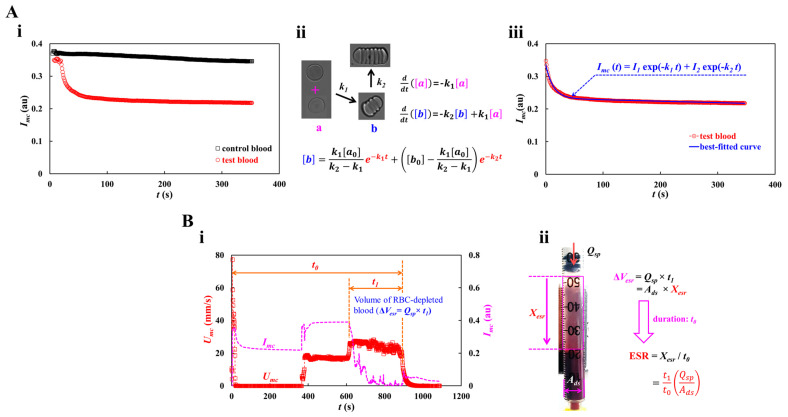
Quantification procedures of RBC aggregation and RBC sedimentation. (**A**) Quantification of RBC aggregation. At the turn-off syringe pump, the *I_mc_* was redraw after clamping the tubing with the pinch valve. (**i**) Temporal variations of *I_mc_* with respect to control blood and test blood. (**ii**) Kinetic representation of isolated and aggregated RBCs. Here, [a] and [b] represent concentration of isolated RBCs and concentration of aggregated RBCs, respectively. *k*_1_ and *k***_2_** denote kinetic constant from isolated RBCs to aggregated RBCs, and kinetic constant between both aggregated RBCs. The analytical solution of [*b*] was derived as $[b]=\frac{k_{1}[a_{0}]}{k_{2}-k_{1}}e^{-k_{1}t}+([b_{0}]-\frac{k_{1}[a_{0}]}{k_{2}-k_{1}})e^{-k_{2}t}$. (**iii**) Regression analysis of *I_mc_*. Taking into account the fact that the [*b*] was proportional to *I_mc_* (i.e., [*b*]~*I_mc_*), the regression formula of *I_mc_* was then assumed as $I_{mc}=I_{1}e^{-k_{1}t}+I_{2}e^{-k_{2}t}$. According to nonlinear regression analysis, four unknown constants were obtained as *I*_1_ = 0.1015 (au), *I*_2_ = 0.2328 (au), *k*_1_ = 0.0767 (1/s), and *k*_2_ = 0.0002 (1/s). (**B**) Quantification procedure of RBC sedimentation. (**i**) Temporal variations of *I_mc_* and *U_mc_*. Here, *t*_0_ and *t*_1_ represented overall delivery time of blood and specific duration of RBC-free blood, respectively. Volume of RBC-depleted blood was calculated as Δ*V_esr_* = *Q_sp_* × *t*_1_. (**ii**) Calculation of RBC sedimentation rate (ESR). The Δ*V_esr_* was calculated as Δ*V_esr_* = *A_ds_* × *X_esr_* = *Q_sp_* × *t*_1_. Here, the *A_ds_* denotes cross-sectional area of driving syringe. The expression of RBC sedimentation was given as $X_{esr}=t_{1}\left(\frac{Q_{sp}}{A_{ds}}\right)$. Considering that the xesr occurred during overall delivery time (*t*_0_), the formula of RBC sedimentation rate was then derived as ESR = $\left(\frac{t_{1}}{t_{0}}\right)\left(\frac{Q_{sp}}{A_{ds}}\right)$ by dividing *X_esr_* by *t*_0_.

The blood image intensity (*I_mc_*) was measured as a function of time (Figure 1D) after turning off the syringe pump and obstructing the Tygon tubing with the pinch valve. Figure 2(Ai) shows the temporal variations in *I_mc_* for the control and test blood. According to a previous study, the maximum concentration is less than 20 mg/mL, considering human physiological and pathological conditions [40]. A dextran solution (20 mg/mL) was selected as the medium for the test blood. The dextran solution contributed substantially to the decrease in image intensity. According to previous studies [39,58], the RBC aggregation process is represented by the summation of two exponential functions. The first and second functions denote the fast and slow aggregation processes, respectively. Recently, a first-order kinetic model was adopted to explain RBC aggregation [41,59]. RBC aggregation using the two exponential functions has not been explained clearly in the literature. Figure 2(Aii) shows the process of RBC aggregation using first-order kinetics). Here, [*a*] and [*b*] represent the concentrations of isolated and aggregated RBCs, respectively. *k*_1_ is the kinetic constant from the isolated RBCs to the aggregated RBCs, and *k*_2_ is the kinetic constant between the two aggregated RBCs. Based on the first-order kinetics of RBC aggregation, two first-order differential equations can be derived as $\frac{d}{dt}([a])=-k_{1}[a]\;$and $\frac{d}{dt}([b])=-k_{2}[b]+k_{1}[a]$. The analytical solution of [*b*] is derived as $[b]=\frac{k_{1}[a_{0}]}{k_{2}-k_{1}}e^{-k_{1}t}+([b_{0}]-\frac{k_{1}[a_{0}]}{k_{2}-k_{1}})e^{-k_{2}t}$, where [*a*_0_] and [*b*_0_] are the initial concentrations of the isolated and aggregated RBCs, respectively. As shown in Figure 2(Ai), RBC aggregation resulted in a decrease in *I_mc_*. Here, it is assumed that [*b*] is proportional to *I_mc_* (i.e., [*b*]~*I_mc_*). The process of RBC aggregation can be represented as $I_{mc}=I_{1}e^{-k_{1}t}+I_{2}e^{-k_{2}t}$. The first-order kinetic model of RBC aggregation is thus expressed as the summation of two exponential functions. Figure 2(Aiii) show the temporal variations in *I_mc_* best fitted with two exponential functions. Nonlinear regression analysis was used to determine the four unknown constants of the regression formula: *I*_1_ = 0.1015 (au), *I*_2_ = 0.2328 (au), *k*_1_ = 0.0767 (1/s), and *k*_2_ = 0.0002 (1/s).

As shown in Figure 2(Bi), the volume of RBC-depleted blood can be estimated using time-lapse *U_mc_* or *I_mc_*. As shown in Figure 1(Bi), the test blood was segregated into two regions: RBC-depleted blood (upper region) and RBC-rich blood (lower region). When the RBC-rich blood flowed into the microfluidic channel, *U_mc_* and *I_mc_* remained unchanged over time. However, when the RBC-depleted blood was loaded into the microfluidic channel, the blood velocity increased substantially. In addition, *I_mc_* decreased significantly and remained near zero over time. Thus, it was possible to detect the volume of RBC-depleted blood from the specific time when *U_mc_* or *I_mc_* changed substantially. Two types of time variables (*t*_0_ and *t*_1_), which denote the overall blood delivery time and specific period of RBC-free blood, respectively, were estimated by inspecting the time-lapse *U_mc_* and *I_mc_*. As test blood was loaded at a constant flow rate (*Q_sp_*), volume of RBC-depleted blood was calculated as Δ*V_esr_* = *Q_sp_* × *t*_1_. As shown in Figure 2(Bii), the RBC sedimentation distance (*X_esr_*) and RBC sedimentation rate (ESR) were estimated in terms of the geometric dimensions of the driving syringe (cross-sectional area: *A_ds_*), two types of time variables (*t*_0_ and *t*_1_), and the flow rate of the syringe pump (*Q_sp_*). The volume of RBC-depleted blood was calculated as Δ*V_esr_* = *A_ds_* × *X_esr_* = *Q_sp_* × *t*_1_. The RBC sedimentation distance was expressed as $X_{esr}=t_{1}\left(\frac{Q_{sp}}{A_{ds}}\right)$. Considering that the sedimentation distance *X_esr_* corresponds to the overall blood delivery time *t*_0_, the RBC sedimentation rate was computed as ESR = $\left(\frac{t_{1}}{t_{0}}\right)\left(\frac{Q_{sp}}{A_{ds}}\right)$ by dividing *X_esr_* by *t*_0_. Table 1 shows all the variables. For the test blood, the *X_esr_* and ESR were estimated to be 20.51 mm and 83.81 mm/h, respectively.

**Figure 3:**
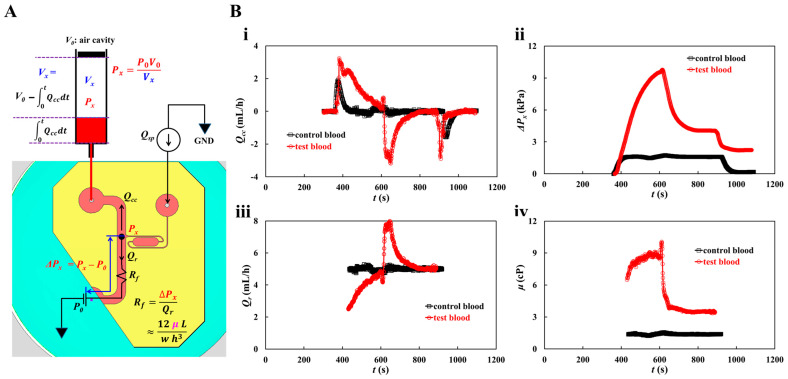
Quantification procedure of blood pressure and blood viscosity using a fluidic circuit model. Blood flow rate set to constant value (*Q_sp_*) with syringe pump. (**A**) Fluidic circuit model of the suggested microfluidic channel. (**B**) Quantification of blood viscosity. When flow rate of syringe pump set to *Q_sp_* = 5 mL/h, the average velocity of each blood was acquired as <*U_mc_*> = 21.8295 mm/s (control blood) and <*U_mc_*> = 17.0582 mm/s (test blood), respectively. With regard to the test blood, the <*U_mc_*> was obtained by averaging *U_mc_* from *t* = 450 s to *t* = 550 s. The flow rate in the compliance channel (*Q_cc_*) was then calculated as *Q_cc_* = (*U_cc_*/<*U_mc_*>) × *Q_sp_*. (**i**) Temporal variations of *Q_cc_* with respect to control blood and test blood. (**ii**) Temporal variations in pressure drop (Δ*P_x_*) with respect to control blood and test blood. (**iii**) Temporal variation of *Q_r_* with respect to control blood and test blood. Here, the *Q_r_* was obtained over the specific time when the *U_mc_* stayed unchanged. (**iv**) Temporal variations in μ with respect to control blood and test blood.

The blood pressure in the first segment of the main channel was determined by analyzing the blood velocity in the compliance channel (*U_cc_*). Based on blood pressure and the Hagen–Poiseuille law, the blood viscosity can be estimated over time. As the syringe pump was set to a constant value (*Q_sp_*), *U_mc_* remained unchanged over time after the transient blood flow elapsed. To derive the blood pressure at the junction (*x*) between the compliance channel and the main channel, as shown in Figure 3A, a simple fluidic circuit model of the suggested microfluidic channel was constructed with constant sources (flow rate of the syringe pump *Q_sp_*, atmospheric pressure *P*_0_, and fluidic resistance *R_f_*). *Q_cc_* and *Q_r_* are the flow rates in the compliance and main channels, respectively. GND (‘▼’) denotes the zero value of pressure. The blood volume in the air damper through the compliance channel was estimated as $\int_{0}^{t}Q_{cc}dt$. The air volume within the air damper decreased from *V_x_* = *V*_0_ to *V_x_* = *V*_0_ − $\int_{0}^{t}Q_{cc}dt$, where *V*_0_ is the initial air cavity inside the air damper. For convenience, it was assumed that pressure drop resulting from blood flow through the compliance channel was negligible. At junction (*x*), the blood pressure is denoted as *P_x_*. Based on the ideal gas law (i.e., pressure × volume = constant), the junction pressure was calculated as $P_{x}=\frac{P_{0}\times V_{0}}{V_{x}}$. The pressure-drop between junction point and reservoir (Δ*P_x_* = *P_x_* − *P*_0_) and *Q_r_* (*Q_r_* = *Q_sp_* − *Q_cc_*) were specified through the first segment of the main channel. Using the Hagen–Poiseuille law, the formula of fluidic resistance was derived as *R_f_* = Δ*P_x_*/*Q_r_*. For a rectangular channel with low aspect ratio (width (*w*) >> depth (*h*)), *R_f_* was approximated as $R_{f}=\frac{12\;\mu \;l}{w\;h^{3}}$, where *l* is the length of the first segment of the main channel. With *w*, *h*, and *l* specified, the fluid viscosity (or blood viscosity) was estimated from the $R_{f}$ expression.

The flow rate of the syringe pump was set to *Q_sp_* = 5 mL/h. As shown in Figure 1(Di), the average velocity of the control blood was estimated to be <*U_mc_*> = 21.8295 mm/s. Considering the test blood, RBC-rich and RBC-depleted blood were loaded into a microfluidic device sequentially. Because RBC-rich blood plays the role of a fluid tracer, blood velocity can be obtained by conducting time-resolved micro-PIV techniques. The average velocity of the test blood was obtained by averaging *U_mc_* from *t* = 450 s to *t* = 550 s. Considering that Hct contributes to reducing the blood velocity obtained by micro-PIV techniques, we inferred that the Hct of RBC-rich blood increased during RBC sedimentation in the driving syringe. *U_cc_* was converted into *Q_cc_* using the formula *Q_cc_* = (*U_cc_*/<*U_mc_*>) × *Q_sp_*. Figure 3(Bi) shows the temporal variations in *Q_cc_* for the control and test blood. The *Q_cc_* of the test blood was higher than that of the control blood, and it gradually decreased and was retained for a longer period. When the RBC-depleted blood was loaded into the main channel, the flow direction reversed and the flow velocity increased significantly. Thereafter, it gradually decreased. Figure 3(Bii) shows the temporal variations in pressure drop (Δ*P_x_*) for the control and test blood. For the control blood, Δ*P_x_* remained unchanged except during the transient periods. By contrast, Δ*P_x_* of test blood increased continuously after the syringe pump was turned on. It decreased significantly when RBC-depleted blood flowed into the main channel. Figure 3(Biii) shows the temporal variations in *Q_r_* for the control and test blood. *Q_r_* was obtained over the specific period when *U_mc_* remained unchanged. The *Q_r_* value of the control blood remained unchanged. By contrast, the *Q_r_* value of the test blood increased gradually until the RBC-rich blood flowed into the main channel. *Q_r_* increased suddenly when the RBC-depleted blood flowed into the main channel; thereafter, it decreased gradually and then remained unchanged over time. Figure 3(Biv) shows the temporal variations in *μ* for the control and test blood. The blood viscosity profiles were similar to those of Δ*P_x_*. RBC sedimentation in the driving syringe for 5 min, contributed to an increase in blood viscosity when compared with the control blood. Blood viscosity had a direct effect on RBC sedimentation in the driving syringe.

### 3.2. Measuring Fluid Viscosity of Glycerin Solution

**Figure 4:**
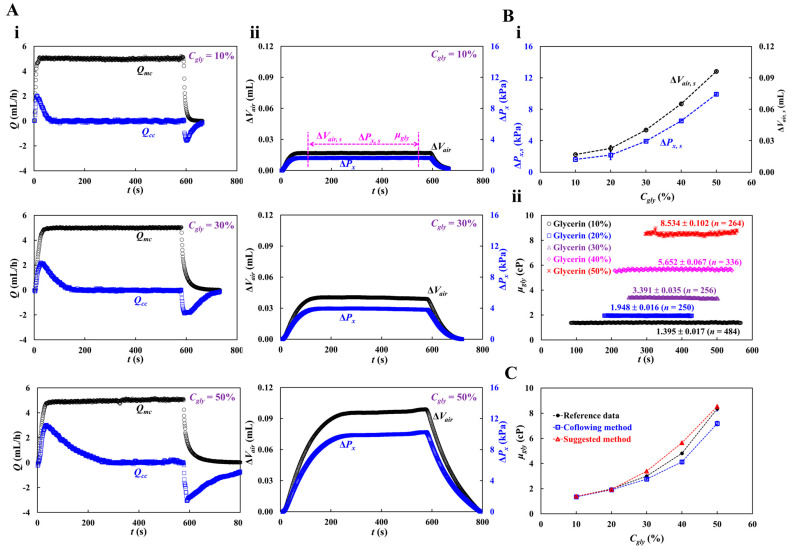
Viscosity measurement with respect to concentration of glycerin solution. Here, flow rate of syringe pump set to 5 mL/h. Shear rate of the flow rate is estimated as $\dot{\gamma }=$3333.3 s^−1^. (**A**) Temporal variations of *Q_mc_* and *Q_cc_* with respect to concentration of glycerin (*C_gly_* = 10%, 30%, and 50%). Here, three parameters (i.e., Δ*V_air,s_*, Δ*P_x,s_*, and *μ_gly_*) were acquired over specific duration while Δ*V_air_* and Δ*P_x_* remained unchanged. (**B**) Quantification of viscosity with respect to *C_gly_* = 10%, 20%, 30%, 40%, and 50%. (**i**) Variations of Δ*V_air,s_* and Δ*P_x,s_* with respect to *C_gly_*. (**ii**) Temporal variations in μ*_Gly_* with respect to *C_gly_*. The corresponding viscosity of each concentration was obtained as *μ_gly_* = 1.395 ± 0.017 cP (*n* = 484, *C_gly_* = 10%), *μ_gly_* = 1.948 ± 0.016 cP (*n* = 250, *C_gly_* = 20%), *μ_gly_* = 3.391 ± 0.035 cP (*n* = 256, *C_gly_* = 30%), *μ_gly_* = 5.652 ± 0.067 cP (*n* = 336, *C_gly_* = 40%), and *μ_gly_* = 8.534 ± 0.102 cP (*n* = 264, *C_gly_* = 50%). (**C**) Quantitative comparison of glycerin viscosity obtained by three kinds of method (i.e., reference experimental data, coflowing method, and suggested method).

**Figure A3:**
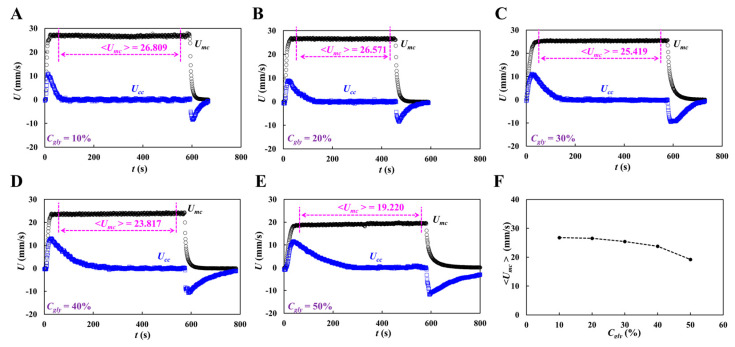
Average velocity of glycerin in the main channel with respect to concentration of glycerin solution (*C_gly_*): (**A**) *C_gly_* = 10%, (**B**) *C_gly_* = 20%, (**C**) *C_gly_* = 30%, (**D**) *C_gly_* = 40%, and (**E**) *C_gly_* = 50%. (**F**) Variations of <*U_mc_*> with respect to *C_gly_*.

To validate the accuracy of fluid viscosity measurement method suggested in this study, a pure glycerin solution was selected as the test fluid. To visualize fluid flow in the main channel, 2% RBC was added as fluid tracer to the glycerin solution. The results were quantitatively compared with those obtained by a previous method (i.e., the coflowing method). The viscosities of various concentrations of glycerin solution were measured at a constant flow rate (*Q_sp_* = 5 mL/h). Based on the shear rate formula of the rectangular channel with low aspect ratio ($\dot{\gamma }=\frac{6\;Q_{sp}}{w\;h^{2}}$) [57], the shear rate of the flow was estimated to be $\dot{\gamma }=$3333.3 s^−1^. Figure A3 (Appendix A) shows the average velocity for various concentrations of glycerin solution. The results indicate that the average velocity obtained using the micro-PIV technique decreased at higher concentrations of the glycerin solution. Based on the average velocity of each glycerin solution, as shown in Figure 4(Ai), the temporal variations in *Q_mc_* and *Q_cc_* were obtained at *C_gly_* = 10, 30, and 50%.

**Figure A4:**
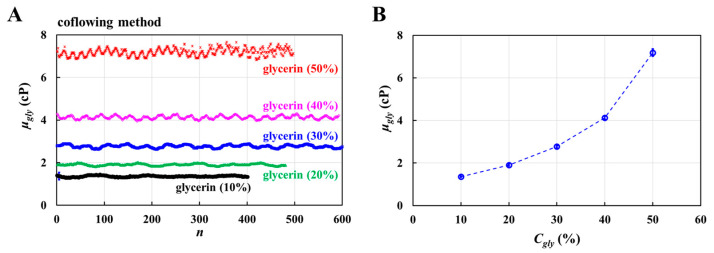
Viscosity obtained by the previous method (coflowing method). (**A**) Temporal variations in viscosity with respect to *C_gly_*. (**B**) Variations of *μ_gly_* with respect to *C_gly_*.

The maximum value of *Q_cc_* increased with *C_gly_*. The transient period of *Q_cc_* increased with *C_gly_*. Figure 4(Aii) shows the temporal variations in Δ*V_air_* at *C_gly_* = 10, 30, and 50%. Three parameters (i.e., Δ*V_air,s_*, Δ*P_x,s_*, and *μ_gly_*) were measured during the specific duration when Δ*V_air_* and Δ*P_x_* remained unchanged. Figure 4(Bi) shows the variations in Δ*V_air,s_* and Δ*P_x, s_* with respect to *C_gly_*. Both tended to increase with *C_gly_*. Figure 4(Bii) shows the temporal variations in *μ_gly_* with respect to *C_gly_*. The corresponding viscosity of each concentration was obtained as *μ_gly_* = 1.395 ± 0.017 cP (*n* = 484, *C_gly_* = 10%), *μ_gly_* = 1.948 ± 0.016 cP (*n* = 250, *C_gly_* = 20%), *μ_gly_* = 3.391 ± 0.035 cP (*n* = 256, *C_gly_* = 30%), *μ_gly_* = 5.652 ± 0.067 cP (*n* = 336, *C_gly_* = 40%), and *μ_gly_* = 8.534 ± 0.102 cP (*n* = 264, *C_gly_* = 50%). Herein, all data of blood rheological data were represented as mean ± standard deviation. For quantitative comparison, as shown in Figure A4 (Appendix A), the coflowing method was adopted to obtain temporal variations in *μ_gly_* with respect to *C_gly_*. For reference, experimental data on the temporal variations in *μ_gly_* with respect to *C_gly_* was obtained [60]. As shown in Figure 4C, the variations in glycerin viscosity obtained using the three approaches (i.e., reference experimental data, coflowing method, and proposed method) overlapped. The viscosity readings were consistent between the proposed method and reference experimental data, whereas the coflowing method underestimated the viscosity of glycerin for *C_gly_* > 40%.

**Figure 5:**
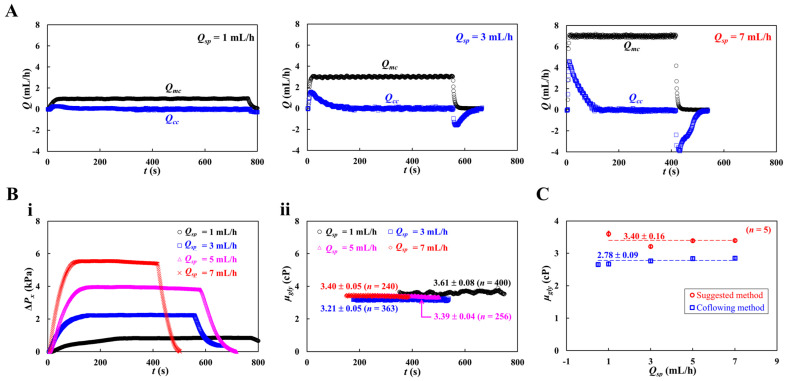
Viscosity measurement of glycerin solution (*C_gly_* = 30%) with respect to flow rate. Here, flow rate of syringe pump set to *Q_sp_* = 1, 3, 5, and 7 mL/h. The shear rate was ranged from $\dot{\gamma }=666.6$ s^−1^ to $\dot{\gamma }=4666.6$ s^−1^. (**A**) Temporal variations of *Q_mc_* and *Q_cc_* with respect to flow rate (*Q_sp_* = 1, 3, and 7 mL/h). (**B**) Quantification of Δ*P_x_* and *μ_gly_.* (**i**) Temporal variations of Δ*P_x_* with respect to *Q_sp_*. (**ii**) Temporal variations in μ*_gly_* with respect to *Q_sp_*. (**C**) Quantitative comparison of glycerin viscosity obtained by two kinds of method (i.e., suggested method and coflowing method). Both methods give constant values of glycerin viscosity with respect to flow rate (i.e., suggested method: *μ_gly_* = 3.40 ± 0.16 cP, coflowing method: *μ_gly_* = 2.78 ± 0.09 cP). The suggested method overestimated viscosity about 17.8% when compared with coflowing method (i.e., previous method).

The viscosity of the glycerin solution (*C_gly_* = 30%), which had a similar value to blood viscosity, was measured with respect to the flow rate of the syringe pump (*Q_sp_*). The flow rate of the syringe pump was set to *Q_sp_* = 1, 3, 5, and 7 mL/h. The shear rate was varied from $\dot{\gamma }=666.6$ s^−1^ to $\dot{\gamma }=4666.6$ s^−1^. Figure 5A shows the temporal variations in *Q_mc_* and *Q_cc_* with respect to the flow rate (*Q_sp_* = 1, 3, and 7 mL/h). When *Q_sp_* increased, the maximum value of *Q_cc_* increased. *Q_cc_* decreased slowly over time after reaching its peak value.

Based on time-lapse *Q_cc_*, variations in Δ*P_x_* and *μ_gl_*_y_ were obtained with respect to *Q_sp_*. Figure 5(Bi) shows the temporal variations in Δ*P_x_* with respect to *Q_sp_*. As expected, *Q_sp_* contributed to an increase in Δ*P_x_* substantially. Figure 5(Bii) shows the temporal variations in *μ_gly_* with respect to *Q_sp_*. The viscosity of the glycerin solution remained constant with respect to the flow rate. Figure 5C shows a quantitative comparison of glycerin viscosity obtained by the two methods (i.e., suggested method, and coflowing method). Both methods gave constant values of glycerin viscosity with respect to the flowrate (suggested method: *μ_gly_* = 3.40 ± 0.16 cP, and coflowing method: *μ_gly_* = 2.78 ± 0.09 cP). The proposed method overestimated the viscosity by approximately 17.8% compared with the coflowing method and by 13.3% compared with the reference experimental data [60].

### 3.3. Measuring Fluid Viscosity of Control Blood with Respect to Hematocrit

After the viscosity measurement of the pure liquid (glycerin solution), suspended blood was used as a complex fluid to validate the performance of the proposed method. To exclude RBC aggregation and sedimentation, 1× PBS was used as the blood medium. To determine the effect of RBC volume (or Hct) on blood viscosity, test blood was prepared by adding normal RBCs to 1× PBS. Hct was adjusted between 20 and 60%. The flow rate of the syringe pump was set to *Q_sp_* = 5 mL/h.

**Figure 6:**
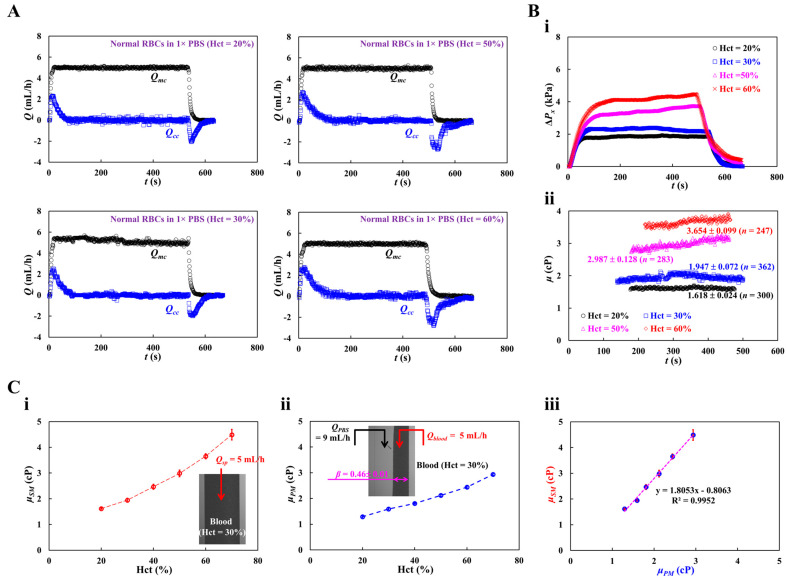
Quantification of blood viscosity of control blood with respect to hematocrit. The hematocrit of control blood was adjusted to Hct = 20%, 30%, 40%, 50%, and 60% by adding normal RBCs into 1× PBS. (**A**) Temporal variations of *Q_mc_* and *Q_cc_* with respect to Hct = 20%, 30%, 50%, and 60%. (**B**) Temporal variations of Δ*P_x_* and *μ* with respect to Hct. (**i**) Temporal variations of Δ*P_x_* with respect to Hct = 20%, 30%, 50%, and 60%. (**ii**) Temporal variations in μ with respect to Hct = 20%, 30%, 50%, and 60%. The corresponding viscosity of each hematocrit was obtained as *μ* = 1.618 ± 0.024 cP (*n* = 300, Hct = 20%), *μ* = 1.947 ± 0.072 cP (*n* = 362, Hct = 30%), *μ* = 2.987 ± 0.128 cP (*n* = 283, Hct = 50%), and *μ* = 3.654 ± 0.099 cP (*n* = 247, Hct = 60%). (**C**) Quantitative comparison of blood viscosity between suggested method and previous method. (**i**) Variations in blood viscosity acquired by the suggested method (*μ_SM_*) with respect to Hct. Inset showed a microscopic image of blood flow (Hct = 30%, flow rate: *Q_sp_* = 5 mL/h).(**ii**) Variations in blood viscosity obtained by the previous method (*μ_PM_*) with respect to Hct. (**iii**) Linear correlation of blood viscosity obtained by both methods. According to regression analysis, both methods give a certain linear relationship (i.e., *μ_SM_* = 1.8053 *μ_PM_* − 0.8063, R^2^ = 0.9952).

Figure 6A shows the temporal variations in *Q_mc_* and *Q_cc_* with respect to Hct = 20, 30, 50, and 60%. Although *Q_mc_* remained constant within steady time intervals, *Q_cc_* varied substantially during the initial transient interval. Based on time-lapse *Q_cc_*, Δ*P_x_*, and *μ* were obtained with respect to Hct. Figure 6(Bi) shows the temporal variations in Δ*P_x_* with respect to Hct = 20, 30, 50, and 60%. The maximum value of Δ*P_x_* increased with Hct percentage. Hct contributed substantially to an increase in the pressure drop. Figure 6(Bii) shows the temporal variations in *μ* with respect to Hct = 20, 30, 50, and 60%. Figure 6(Ci,ii) show the variations in blood viscosity obtained using the proposed method (*μ_SM_*) and previous method (*μ_PM_*) with respect to Hct. The proposed method overestimated the viscosity of the control blood compared with the previous method (i.e., the coflowing method). As shown in the inset of Figure 6(Cii), with regard to the coflowing method, the corresponding flow rates of the test blood (Hct = 30%) and 1× PBS were adjusted to *Q_blood_* = 5 mL/h and *Q_PBS_* = 9 mL/h, respectively. The coflowing channel was partially filled with blood (volume fraction = 46%) and 1× PBS (volume fraction = 54%). However, the main channel was filled with blood (volume fraction = 100%) at a flow rate of *Q_sp_* = 5 mL/h. We inferred that blood volume might influence blood viscosity measurement (i.e., Fåhraeus–Lindqvist effect) [61,62,63]. That is, the difference in the blood filling volume substantially contributed to the change in blood viscosity. Next, we measured the correlation of blood viscosity obtained by the two methods. Figure 6 (Ciii) shows the blood viscosity obtained using the previous method (*μ_PM_*) on the X-axis and the blood viscosity obtained using the suggested method (*μ_SM_*) on the Y-axis. Linear regression analysis was performed to obtain a linear relationship: *μ_SM_* = 1.8053 *μ_PM_* – 0.8063, R^2^ = 0.9952. The high value of R^2^ confirmed that the proposed method can be used to measure blood viscosity with consistency when compared with the coflowing method.

### 3.4. Measuring Multiple Rheological Properties of Dextran-Induced Blood

To determine the effect of RBC sedimentation in the driving syringe on blood viscosity measurement in a microfluidic device, dextran solution (*C_dex_* = 5, 10, 15, and 20 mg/mL), which accelerates RBC sedimentation in the driving syringe, was selected as the blood medium [64,65,66,67]. Furthermore, to stimulate RBC sedimentation in the driving syringe, the Hct of the test blood was set to 30% [53,65]. The test blood was prepared by adding normal RBCs to each dextran solution concentration.

**Figure 7:**
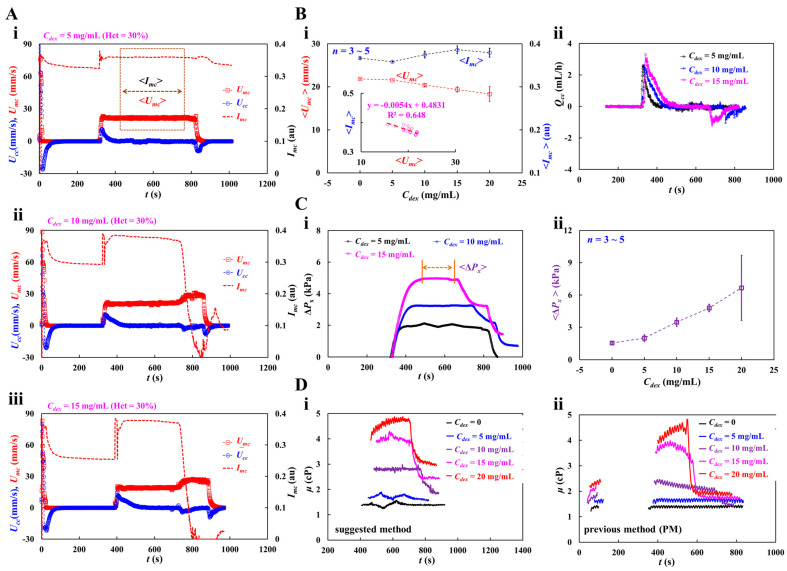
Blood viscosity measurement of dextran-induced blood. To induce RBC sedimentation in the driving syringe, dextran solution (*C_dex_* = 5, 10, 15, and 20 mg/mL) was selected as blood medium. Hematocrit of test blood was adjusted to Hct = 30% by adding normal RBCs into the specific dextran solution. (**A**) Temporal variations of *U_mc_*, *U_cc_*, and *I_mc_* with respect blood medium (*C_dex_*): (**i**) *C_dex_* = 5 mg/mL, (**ii**) *C_dex_* = 10 mg/mL, and (**iii**) *C_dex_* = 15 mg/mL. Here, the average value of blood velocity and image intensity (<*U_mc_*>, <*I_mc_*>) in the main channel was calculated by averaging *U_mc_* and *I_mc_* over the specific duration when they both remained unchanged. (**B**) Contribution of dextran solution to <*U_mc_*>, <*I_mc_*> and *Q_cc_*. (**i**) Variations of <*U_mc_*> and <*I_mc_*> with respect to *C_dex_* = 0, 5, 10, 15, and 20 mg/mL. The inset liner relationship between <*I_mc_*> and <*U_mc_*>. Linear regression formula was obtained as <*I_mc_*> = −0.0054 <*U_mc_*> + 0.4831 (R^2^ = 0.648). (**ii**) Temporal variations of *Q_cc_* with respect to *C_dex_*. (**C**) Variations in blood pressure (Δ*P_x_*) with respect to blood medium (*C_dex_*). (**i**) Temporal variations of Δ*P_x_* with respect to *C_dex_* = 5, 10, and 15 mg/mL. Average value of Δ*P_x_* (<Δ*P_x_*>) was calculated over the time duration when the Δ*P_x_* was unchanged and denoted as “←→”. (**ii**) Variations of <Δ*P_x_*> with respect to *C_dex_* = 0, 5, 10, 15, 20 mg/mL. (**D**) Quantitative comparison between suggested method and previous method. (**i**) Temporal variations in μ obtained by the suggested method with respect to *C_dex_*. (**ii**) Temporal variations in μ obtained by the previous method with respect to *C_dex_*.

Figure 7A shows the temporal variations in *U_mc_*, *U_cc_*, and *I_mc_* with respect to the concentration of the dextran solution (*C_dex_* = 5, 10, and 15 mg/mL). The average values of blood velocity and image intensity (<*U_mc_*> and <*I_mc_*>) in the main channel were calculated by averaging *U_mc_* and *I_mc_* over the specific duration when they both remained unchanged. When the blood flow was stopped in the main channel, *I_mc_* decreased substantially with respect to *C_dex_*. The RBC aggregation resulting from the dextran solution contributed to a decrease in *I_mc_*. When the test blood was loaded into the main channel, *I_mc_* and *U_mc_* remained constant for a certain duration. However, when the RBC-depleted blood flowed into the main channel, *U_mc_* increased and *I_mc_* decreased significantly. The experimental investigation revealed that the dextran solution increased RBC sedimentation in the driving syringe, and consequently, the delivery time of the RBC-depleted blood was longer at higher *C_dex_*.

Under steady blood flow in the main channel, as shown in Figure 7(Bi), the variations in <*U_mc_*> and <*I_mc_*> were measured with respect to *C_dex_* = 0, 5, 10, 15, and 20 mg/mL. Even though the blood flow rate remained constant, <*U_mc_*> decreased gradually with increasing *C_dex_*. However, *I_mc_* did not exhibit any specific trend with respect to *C_dex_*. The inset of Figure 7(Bi) shows the linear regression equation: <*I_mc_*> = −0.0054 <*U_mc_*> + 0.4831 (R^2^ = 0.648). Figure 7(Bii) shows the temporal variations in *Q_cc_* with respect to *C_dex_*. A higher concentration of the dextran solution contributed to increasing variations in *Q_cc_* at the transient flow. In addition, the dextran solution shortened the time when the flow is reversed in the compliance channel (i.e., *Q_cc_* < 0).

Figure 7(Ci) shows the variations in Δ*P_x_* with respect to *C_dex_* = 5, 10, and 15 mg/mL. The dextran solution increased Δ*P_x_* significantly. When the RBC-depleted blood entered the main channel, Δ*P_x_* decreased over time substantially. <Δ*P_x_*> was calculated by averaging Δ*P_x_* over the specific duration when it remained unchanged. Figure 7(Cii) shoes the variations in <Δ*P_x_*> with respect to *C_dex_* = 0, 5, 10, 15, 20 mg/mL. <Δ*P_x_*> increased significantly at higher *C_dex_*. At *C_dex_* = 20 mg/mL, <Δ*P_x_*> exhibited relatively large fluctuations.

Figure 7(Di) shows the temporal variations in *μ* obtained using the suggested method with respect to *C_dex_*. When the concentration of the dextran solution was less than 10 mg/mL, *μ* remained constant over time. At *C_dex_* = 10 mg/mL, RBC-depleted blood showed a substantial reduction in blood viscosity. However, when the concentration of the dextran solution was > 15 mg/mL, *μ* exhibited some fluctuations. The viscosity of RBC-depleted blood decreased substantially compared with that of RBC-rich blood. The blood viscosity results obtained using the proposed method were compared with those using a previous method (i.e., coflowing method). For the coflowing method, two syringe pumps were employed; all other conditions were identical to those for the proposed method. Figure 7(Dii) shows the temporal variations in *μ* obtained using the coflowing method with respect to *C_dex_*. The results were similar to those obtained using the proposed method. The coflowing method underestimated the blood viscosity in compared to the proposed method. After RBC sedimentation for 5 min, blood viscosity increased significantly in comparison to the blood viscosity during the initial period. The experimental results showed that RBC sedimentation in the driving syringe contributed to a significant change in blood viscosity. In the proposed method, RBC aggregation occurred when the blood flow from the compliance channel to the air damper was stopped. The Hct of the RBC-rich blood was not uniformly distributed in the compliance channel. When the RBC-depleted blood entered the main channel, blood flow reversed in the compliance channel (i.e., from ‘+’ sign to ‘-’ sign). The blood Hct in the compliance channel was substantially different from that in the RBC-rich blood. The micro-PIV technique did not provide an accurate blood flow rate in the compliance channel because its accuracy was substantially influenced by blood hematocrit. For this reason, the proposed method significantly overestimated the viscosity of RBC-depleted blood compared with the coflowing method. The coflowing method yielded consistent results without Hct variations, wherein the flow rates of both fluids were maintained at a constant rate.

**Figure 8:**
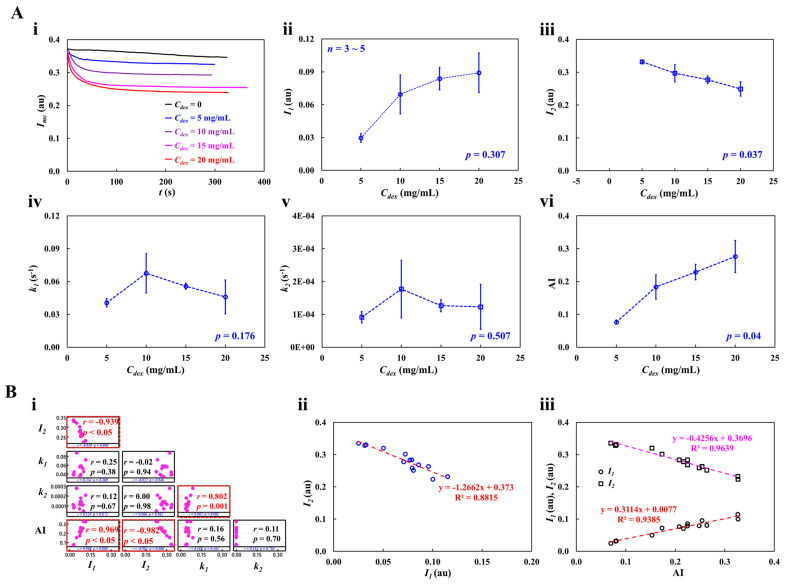
RBC aggregation measurement of dextran-included blood (Hct = 30%). (**A**) Quantification of RBC aggregation with respect to concentration of dextran solution. (**i**) Temporal variations of *I_mc_* with respect to *C_dex_* = 0, 5, 10, 15, and 20 mg/mL. Based on time-lapse image intensity (*I_mc_*), four unknown variables (*I*_1_, *I*_2_, *k*_1_, and *k*_2_) of regression formula ($I_{mc}=I_{1}e^{-k_{1}t}+I_{2}e^{-k_{2}t}$) were acquired by conducting nonlinear regression analysis. (**ii**) Variations of *I*_1_ with respect to *C_dex_* (*p* = 0.307). (**iii**) Variations of *I*_2_ with respect to *C_dex_* (*p* = 0.037). (**iv**) Variations of *k*_1_ with respect to *C_dex_* (*p* = 0.176). (**v**) Variations of *k*_2_ with respect to *C_dex_* (*p* = 0.507). (**vi**) Variations in AI with respect to *C_dex_* (*p* = 0.04). (**B**) Quantitative comparison between suggested four variables and conventional aggregation index. (**i**) Correlation map among five variables (*I*_1_, *I*_2_, *k*_1_, *k*_2_, and AI). *I*_1_ and *I*_2_ had substantial correlation (*p* < 0.05). The AI exhibited a certain relationship with respect to *I*_1_ and *I*_2_ (*p* < 0.05). (**ii**) Linear relationship between *I*_1_ and *I*_2_. According to linear regression analysis, regression formula (i.e., red-color dash line) was obtained as *I*_2_ = −1.2662 *I*_1_ +0.373 (R^2^ = 0.8815). (**iii**) Linear relation of *I*_1_ and *I*_2_ with respect to AI. Two linear regression formulas (i.e., *I*_1_: red-color dash line, *I*_2_: purple-color dash line) were obtained as *I*_1_ = 0.3114 AI + 0.0077 (R^2^ = 0.9383) and *I*_2_ = −0.4256 AI + 0.3696 (R^2^ = 0.9639).

As shown in Figure 8(Ai), at the stoppage of blood flow in the main channel, the time-lapse *I_mc_*, which was used to quantify RBC aggregation, was redrawn with *C_dex_* = 0, 5, 10, 15, and 20 mg/mL. The results indicated that *I_mc_* variations were larger at higher concentrations of the dextran solution. To quantify RBC aggregation as a function of dextran solution concentration, the time-lapse *I_mc_* was best fitted as $I_{mc}=I_{1}e^{-k_{1}t}+I_{2}e^{-k_{2}t}$. The four constants of the regression formula were estimated through nonlinear regression analysis using EXCEL^TM^ (Office 365, Microsoft, Redmond, Washington, DC, USA). Figure 8(Aii) shows the variation in *I*_1_ with respect to *C_dex_*. *I*_1_ increased gradually with respect to *C_dex_*. The statistical software (Minitab ver. 21, State College, PA, USA) and statistical significance was determined through the one-way ANOVA test. The *I*_1_ variations with respect to *C_dex_* were not statistically significant (*p* = 0.307).

Figure 8(Aiii) shows the variation in *I*_2_ with respect to *C_dex_*. The *I*_2_ variations with respect to *C_dex_* were statistically significant (*p* = 0.037). Figure 8 (Aiv,v) show plots of *k*_1_ and *k*_2_ with respect to *C_dex_*. *k*_1_ and *k*_2_ did not exhibit significant differences with respect to *C_dex_* (*p* = 0.176 for *k*_1_, *p* = 0.507 for *k*_2_). To compare the four parameters obtained using the proposed method, as shown in Figure 8(Avi), variations in AI were measured with respect to *C_dex_*. AI increased gradually with *C_dex_*. According to the statistical test, the dextran solution contributed substantially to the increase in the AI (*p* = 0.04). To determine the correlations between *I*_1_, *I*_2_, *k*_1_, *k*_2_, and AI, a correlation map was drawn as a lower triangular matrix: Figure 8(Bi). The four variables (*I*_1_, *k*_1_, *k*_2_, and AI) are positioned in the vertical column. The four variables (*I*_1_, *I*_2_, *k*_1_, and *k*_2_) are positioned in the horizontal row. The correlation between two variables (one variable in the vertical column and the other in the horizontal row) is expressed as *r* (correlation coefficient) and *p*. *I*_1_ and *I*_2_ were significantly correlated (*p* < 0.05). AI was significantly correlated with *I*_1_ and *I*_2_ (*p* < 0.05). AI was not significantly correlated with *k*_1_ and *k*_2_ (*p* > 0.05). Figure 8(Bii) shows the linear regression formula between *I*_1_ and *I*_2_: *I*_2_ = −1.2662 *I*_1_ + 0.373 (R^2^ = 0.8815). Figure 8(Biii) shows the linear regression formulae between AI and *I*_1_ and between AI and *I*_2_: *I*_1_ = 0.3114 AI + 0.0077 (R^2^ = 0.9383) and *I*_2_ = −0.4256 AI + 0.3696 (R^2^ = 0.9639). Based on these results, AI was linearly proportional to *I*_1_ and *I*_2_. Both *I*_1_ and *I*_2_ showed a consistent trend of RBC aggregation when compared to AI.

**Figure 9:**
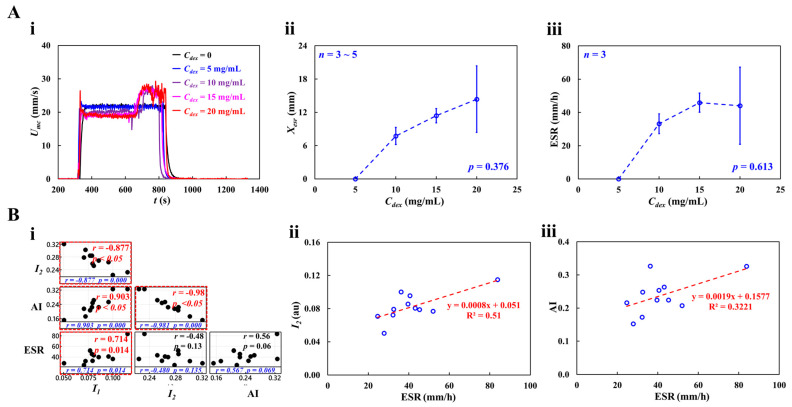
RBC sedimentation measurement of dextran-included blood (Hct = 30%). (**A**) Quantification of RBC sedimentation with respect to concentration of dextran solution. (**i**) Temporal variations in Umc with respect to *C_dex_* = 0, 5, 10, 15, and 20 mg/mL. (**ii**) Variations of *X_esr_* with respect to *C_dex_* (*p* = 0.376). (**iii**) Variations in ESR with respect to *C_dex_* (*p* = 0.613). (**B**) Quantitative comparison between RBC aggregation (*I*_1_, *I*_2_, and AI) and RBC sedimentation (ESR). (**i**) Correlation map among four variables (i.e., *I*_1_, *I*_2_, AI, and ESR). ESR exhibited substantial relationship with respect to *I*_1_ (*p* = 0.014) and AI (*p* = 0.069). (**ii**) Linear relationship between *I*_2_ and ESR. The linear regression (i.e., red-color dash line) was acquired as *I*_2_ = 0.0008 ESR + 0.051 (R^2^ = 0.51). (**iii**) Linear relationship between AI and ESR. The linear regression (i.e., red-color dash line) was acquired as AI = 0.0019 ESR + 0.1577 (R^2^ = 0.3221).

Figure 9(Ai) shows the RBC sedimentation in the driving syringe obtained by analyzing the time-lapse *U_mc_* with respect to *C_dex_*. *U_mc_* remained constant in steady blood flow for *C_dex_* = 0 and 5 mg/mL. However, at *C_dex_* > 10 mg/mL, *U_mc_* increased abruptly when the RBC-depleted blood entered the main channel. Figure 9(Aii) shows the variations in *X_esr_* with respect to *C_dex_*. *X_esr_* tended to increase gradually with *C_dex_* = 10, 15, and 20 mg/mL. *X_esr_* was not statistically correlated with *C_dex_* (*p* = 0.376). Interestingly, *X_esr_* showed large fluctuations at *C_dex_* = 20 mg/mL. Below *C_dex_* = 20 mg/mL, *X_esr_* varied substantially with *C_dex_* (*p* = 0.035).

Figure 9(Aiii) shows the variations in ESR with respect to *C_dex_*. ESR increased gradually up to *C_dex_* = 15 mg/mL (*p* = 0.056). ESR saturated between *C_dex_* = 15 mg/mL and 20 mg/mL. These results were consistent with those of a previous study [65]. Four variables (RBC aggregation: *I*_1_, *I*_2_, and AI; and RBC sedimentation: ESR) were selected to investigate the correlation between RBC aggregation and sedimentation; Figure 9(Bi) shows the correlation map. The three variables (*I*_1_, AI, and ESR) are positioned in the vertical column. The three variables (*I*_1_, *I*_2_, and AI) are positioned in the horizontal row. Statistical analysis showed that ESR was statistically correlated with *I*_1_ (*p* = 0.014) and AI (*p* = 0.069). Figure 9(Bii) shows the linear relationship between *I*_2_ and ESR; the linear regression formula was calculated as *I*_2_ = 0.0008 ESR + 0.051 (R^2^ = 0.51). Figure 9(Biii) shows the linear relationship between AI and ESR: AI = 0.0019 ESR + 0.1577 (R^2^ = 0.3221). The low value of the coefficient of linear regression (R^2^) indicated that RBC aggregation may not be linearly proportional to RBC sedimentation (ESR). By contrast, RBC sedimentation (ESR) strongly correlated with RBC aggregation (*I*_1_ and AI).

### 3.5. Impact of Hematocrit in Dextran-Induced Blood on Blood Rheological Properties

With respect to dextran-containing blood, Hct might influence the multiple rheological properties of blood. The effect of Hct on blood properties was evaluated by adjusting Hct to the same concentration as that of the dextran solution. To significantly accelerate RBC sedimentation in the driving syringe, a dextran solution (20 mg/mL) was used as the blood medium. The Hct of the test blood was adjusted to 20, 30, 40, 50, and 60% by adding normal RBCs to the blood medium.

**Figure 10:**
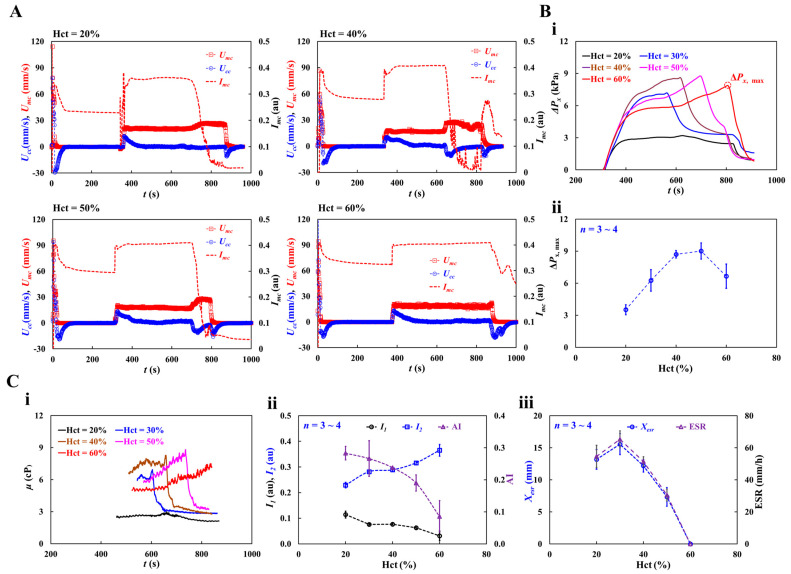
Impact of hematocrit in dextran-included blood on hemorheological properties. Hematocrit of test blood was adjusted to Hct = 20%, 30%, 40%, 50%, and 60% by adding normal RBCs into dextran solution (20 mg/mL). (**A**) Temporal variation of *U_mc_*, *U_cc_*, and *I_mc_* with respect to Hct = 20%, 40%, 50%, and 60%. (**B**) Contribution of hematocrit to blood pressure. (**i**) Temporal variation of Δ*P_x_* with respect to Hct. The Δ*P_x,max_* denotes maximum value of Δ*P_x_*. (**ii**) Variations in maximum pressure (Δ*P_x,max_*) with respect to Hct. (**C**) Quantification of three rheological properties with respect to hematocrit. (**i**) Temporal variations in μ with respect to Hct. (**ii**) Variations of *I*_1_, *I*_2_, and AI with respect to Hct. (**iii**) Variations of *X_esr_* and ESR with respect to Hct.

**Figure A5:**
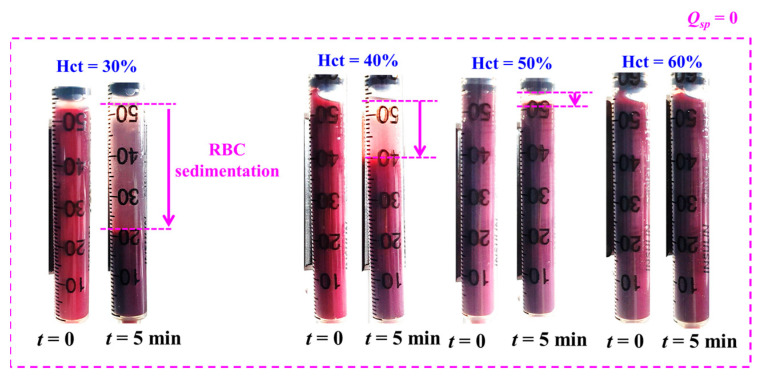
RBC sedimentation in driving syringe with respect to hematocrit. Herein, test blood was prepared by adding normal RBCs into dextran solution (20 mg/mL). At the stoppage of blood flow (*Q_sp_* = 0), after an elapse of 5 min, RBC sedimentation contributed to moving interface between liquid and RBCs along the gravitational direction. Herein, RBC sedimentation denotes purple-arrow. From the results, RBC sedimentation tended to decrease gradually from Hct = 30% to Hct = 50%. RBC sedimentation was not detected with respect to Hct = 60%.

As shown in Figure A5 (Appendix A), RBC sedimentation in the driving syringe was detected with respect to the Hct. At the stoppage of blood flow (*Q_sp_* = 0), after 5 min, RBC sedimentation contributed to the moving interface between the liquid and RBCs in the direction of gravity. The results show that the interface movement decreased from Hct = 30% to Hct = 50%. However, this was not clearly detected for Hct = 60%. Figure 10A shows the temporal variations in *U_mc_*, *U_cc_*, and *I_mc_* with Hct = 20, 40, 50, and 60%. At the stoppage of the blood flow, the variations in *I_mc_* decreased substantially. Variations in *I_mc_* were evident even at Hct = 60%.

Based on the time-lapse *U_cc_*, the contribution of Hct to blood pressure was obtained. Figure 10(Bi) shows the temporal variations in Δ*P_x_* with respect to Hct. Δ*P_x, max_* denotes the maximum value of Δ*P_x_*. The results indicated that Δ*P_x_* increased with Hct. Δ*P_x_* took longer to reach its maximum at higher concentrations of Hct. Except at Hct = 60%, Δ*P_x_* decreased abruptly when the RBC-depleted blood entered the main channel. Figure 10(Bii) shows the variations in Δ*P_x,max_* with respect to Hct. Δ*P_x, max_* increased up to Hct = 50%. At Hct = 60%, Δ*P_x, max_* decreased substantially. As shown in Figure A5 (Appendix A), the blood (Hct = 60%) did not exhibit RBC sedimentation in the driving syringe. The RBC-depleted blood did not enter the main channel which did not contribute to an increase in the hematocrit. Based on time-lapse Δ*P_x_*, Figure 10(Ci) shows the temporal variations in *μ* with respect to Hct. The blood viscosity increased over time. When the RBC-depleted blood flowed in the main channel, the *μ* decreased substantially and remained constant over time. Because RBC sedimentation in the driving syringe contributed to an increase in the Hct, the maximum viscosity of blood with Hct = 40% or 50% was higher than that of blood with Hct = 60%. In other words, RBC sedimentation substantially increases blood viscosity over time. As the RBC-depleted blood flowed into the main channel, blood viscosity decreased substantially and remained constant for a certain duration. Figure 10(Cii) shows the variations in *I*_1_, *I*_2_, and AI with respect to Hct. *I*_1_ and *I*_2_ exhibited substantial variations with Hct. AI decreased gradually with increasing Hct concentration. These results show that *I*_1_, *I*_2_, and AI can be used to effectively detect RBC aggregation. Figure 10(Ciii) shows the variations in *X_esr_* and ESR with respect to Hct. *X_esr_* and ESR decreased substantially with increasing Hct concentration. At Hct = 60%, no RBC sedimentation occurred (*X_esr_* = 0, ESR = 0). Thus, RBC sedimentation was significantly influenced by Hct concentration.

### 3.6. Detection of Hardened RBCs with Multiple Rheological Properties

The proposed method was used to quantify the contribution of RBC deformability to multiple rheological properties of blood. For blood sample preparation, normal RBCs were hardened by exposure to glutaraldehyde solution. To adjust RBC deformability appropriately, normal RBCs were exposed to four concentrations of glutaraldehyde solution (*C_ga_* = 0, 1, 2, and 4 μL/mL). To induce the maximum levels of RBC aggregation (or RBC sedimentation), the hematocrit and blood media were selected as Hct = 30% and dextran solution (20 mg/mL), respectively. The test blood (Hct = 30%) was prepared by adding the hardened RBCs to the specific blood medium.

**Figure 11:**
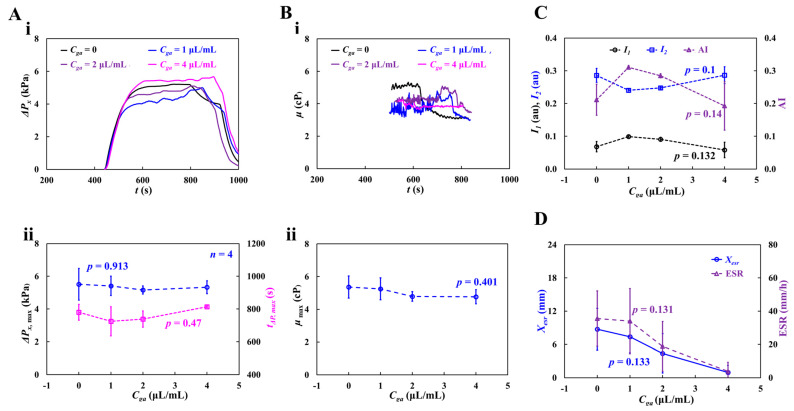
Contributions of partially hardened RBCs to hemorheological properties. The degree of rigidity in RBCs varied by increasing concentration of glutaraldehyde solution ranging from *C_ga_* = 1 μL/mL to *C_ga_* = 4 μL/mL. The test blood (Hct = 30%) was then prepared by adding hardened RBCs into dextran solution (20 mg/mL). (**A**) Variations in blood pressure with respect to *C_ga_*. (**i**) Temporal variations of Δ*P_x_* with respect to *C_ga_*. (**ii**) Variations of Δ*P_x,max_* (*p* = 0.903) and *t*_Δ*p,max*_ (*p* = 0.47) with respect to *C_ga_*. (**B**) Variations in viscosity with respect to *C_ga_*. (**i**) Temporal variations in μ with respect to *C_ga_*. (**ii**) Variations in maximum viscosity (*μ_max_*) (*p* = 0.401). (**C**) Variations of *I*_1_, *I*_2_, and AI with respect to *C_ga_* (*p* = 0.1~0.14). (**D**) Variations of *X_esr_* and ESR with respect to *C_ga_* (*p* = 0.131~0.133).

Figure 11(Ai) shows the temporal variations in blood pressure (Δ*P_x_*) with respect to *C_ga_*. The dynamic variations in Δ*P_x_* did not show any consistent trends with respect to the concentration of glutaraldehyde solution. The maximum value of Δ*P_x_* was not significantly different for *C_ga_* = 0, 1, and 2 μL/mL. The largest Δ*P_x_* was observed for the blood sample with hardened RBCs (*C_ga_* = 4 μL/mL). The specific time (*t*_Δ*p,max*_) when Δ*P_x_* reached its maximum was longer at higher concentrations of the glutaraldehyde solution. That is, the RBC-depleted blood entered the main channel later at higher concentrations of the glutaraldehyde solution. These results indicated that RBC sedimentation decreased at higher concentrations of the glutaraldehyde solution. Figure 11(Aii) shows the variations in Δ*P_x,max_* and *t*_Δ*p,max*_ with respect to *C_ga_*. According to the one-way ANOVA test, Δ*P_x,max_* remained unchanged with respect to *C_ga_* (*p* = 0.903). *t*_Δ*p,max*_ increased from *C_ga_* = 1 μm/mL to *C_ga_* = 4 μm/mL (*p* = 0.47).

Figure 11(Bi) shows the temporal variations in *μ* with respect to *C_ga_*. Compared with normal blood (*C_ga_* = 0), the viscosity of hardened blood decreased with increasing *C_ga_*. At *C_ga_* = 1 or 2 μL/mL, the blood viscosity increased gradually over time until the RBC-depleted blood entered the main channel. At *C_ga_* = 4 μL/mL, the blood viscosity remained constant over time. Because RBC sedimentation in the driving syringe did not occur, Hct of the test blood (*C_ga_* = 4 μL/mL) stayed unchanged during the blood-loading period. Figure 11(Bii) shows the variations in maximum viscosity (*μ_max_*) with respect to *C_ga_*. *μ_max_* decreased with increasing *C_ga_*. Owing to the large fluctuations, the one-way ANOVA test did not show a substantial difference with respect to *C_ga_* (*p* = 0.401).

To quantify RBC aggregation, Figure 11C shows the variations in *I*_1_, *I*_2_, and AI with respect to *C_ga_*. *I*_1_ and AI gradually decreased from *C_ga_* = 1 μL/mL to *C_ga_* = 4 μL/mL. *I*_2_ increased from *C_ga_* = 1 μL/mL to *C_ga_* = 4 μL/mL. From statistical results, the corresponding *p* of each variable was obtained as *p* = 0.132 (*I*_1_), *p* = 0.1 (*I*_2_), and *p* = 0.14 (AI). The variations in RBC aggregation with respect to *C_ga_* were not statistically significant (*p* = 0.132 for *I*_1_, *p* = 0.1 for *I*_2_, and *p* = 0.14 for AI).

To assess the contribution of gradually hardened RBCs to RBC sedimentation, Figure 11D shows the variations in *X_esr_* and ESR with respect to *C_ga_*. *X_esr_* and ESR decreased with increasing *C_ga_*. That is, the lower the RBC deformability, the less RBC sedimentation. The *p-*values were: *p* = 0.133 (*X_esr_*) and *p* = 0.131 (ESR). The experimental results indicate that hardened RBCs potentially contribute to varying rheological properties. Interestingly, most of the rheological properties showed large fluctuations due to the unstable blood flow in the microfluidic channel.

The experimental results showed that the three rheological properties (RBC aggregation, blood viscosity, and RBC sedimentation) can be successfully measured using the proposed method. Furthermore, the proposed method can detect the contribution of RBC sedimentation to blood pressure and viscosity. Future studies should aim to improve the accuracy of measuring the blood flow rate in the compliance channel when the blood is changed from RBC-rich to RBC-depleted in the main channel. However, the performance of the suggested method was validated using suspended blood by changing the hematocrit or blood medium, which were supplied by a blood bank. To impact the present method on the clinical relevance and potential diagnostic applications, the performance of the proposed method should be reevaluated using patient blood supplied from clinics. Furthermore, several blood properties had been measured in room temperature. It was expected that working fluid temperature in the microfluidic chip might exhibit fluctuations over time. Thus, the temperature fluctuation contributes to deteriorate reproducibility and reliability of blood rheological properties. For the reason, to resolve the issue, in a future work, fluid temperature control unit will be integrated into a microfluidic chip.

## 4. Conclusions

In this study, a novel method is proposed and demonstrated to investigate the contribution of RBC sedimentation in a driving syringe to blood microrheology using a microfluidic chip. A single syringe pump was used to infuse the test blood into the microfluidic channel at a constant blood flow rate (*Q_sp_*). The syringe pump was turned off for 5 min to induce RBC sedimentation in the driving syringe. During this period, RBC aggregation was determined by analyzing the time-lapse blood image intensity at stasis in the microfluidic channel. Using a first-order kinetic model, the concentration of aggregated RBC was analytically derived as $[b]=\frac{k_{1}[a_{0}]}{k_{2}-k_{1}}e^{-k_{1}t}+([b_{0}]-\frac{k_{1}[a_{0}]}{k_{2}-k_{1}})e^{-k_{2}t}$. The four variables (*I*_1_, *I*_2_, *k*_1_, *k*_2_) for representing RBC aggregation were then estimated by best fitting the time-lapse intensity as *I*(*t*) = *I*_1_ exp (−*k*_1_*t*) + *I*_2_ exp (−*k*_2_*t*). After 5 min, RBC sedimentation contributed to the separation of test blood into RBC-depleted and RBC-rich blood. RBC-rich and RBC-depleted blood were sequentially infused into the microfluidic chip. With regard to the blood flow rate in the compliance channel (*Q_cc_*), considering that the same blood entered the compliance channel from the main channel and the velocity of the main channel remained constant over time (<*U_mc_*>~*Q_sp_*), the blood velocity in the compliance channel (*U_cc_*) was compensated into the blood flow rate in the compliance channel (*Q_cc_* = *U_cc_*/<*U_mc_*> × *Q_sp_*). Based on the ideal gas law (air pressure × air cavity = constant), the blood pressure was estimated by analyzing the time-lapse blood flow rate in the compliance channel connected to the air damper component. Blood viscosity was obtained using the Hagen–Poiseuille law. When the RBC-depleted blood was loaded into the microfluidic channel, the blood viscosity or image intensity exhibited substantial changes over time. Either the time-lapse blood velocity or image intensity was used to detect RBC sedimentation in the driving syringe. The RBC sedimentation distance (*X_esr_*) and RBC sedimentation rate (ESR) were calculated as $X_{esr}=t_{1}\left(\frac{Q_{sp}}{A_{ds}}\right)$ and ESR *=* $\left(\frac{t_{1}}{t_{0}}\right)\left(\frac{Q_{sp}}{A_{ds}}\right)$, respectively. The performance of the proposed protocols was validated through several experiments using glycerin solution and suspended blood. The viscosities of pure liquid (glycerin) and control blood (Hct = 20–60%, medium: 1× PBS) were measured and compared with the coflowing and the proposed methods. The viscosity obtained using the proposed method was substantially overestimated compared to that obtained using the coflowing method. However, the viscosities obtained using the two methods exhibited a sufficient correlation. Using dextran-containing test blood (dextran concentration: 0~20 mg/mL, Hct = 20~60%), the proposed method was employed to quantify three rheological properties (blood viscosity, RBC aggregation, and RBC sedimentation). Owing to RBC sedimentation in the driving syringe, RBC-rich and RBC-depleted blood were infused sequentially from the driving syringe. The blood viscosity increased substantially over time. It decreased abruptly when the RBC-depleted blood began to flow into the main channel. The time-lapse blood viscosity obtained using the proposed method showed consistent trends when compared with the coflowing method. Regarding RBC aggregation, two of the four variables (*I*_1_, *I*_2_) were strongly correlated with AI. *I*_1_ and *I*_2_ exhibited substantial variations with respect to dextran concentration and Hct. Both indices (*X_esr_*, ESR) representing RBC sedimentation also showed consistent trends with respect to dextran concentration and Hct. The several properties suggested in this study were used to detect partially hardened RBCs. The results indicate that hardened RBCs potentially contribute to variations in the rheological properties of blood. Most of the rheological properties showed large fluctuations owing to the unstable blood flow in the microfluidic channel. In conclusion, the suggested parameters related to the multiple rheological properties of blood (RBC aggregation, blood viscosity, and RBC sedimentation) are effective for monitoring the contribution of RBC sedimentation to multiple rheological properties. A distinctive advantage of the proposed method is that it can measure multiple blood rheological properties simultaneously using a single syringe pump. In addition, it can detect RBC sedimentation in the driving syringe by analyzing the blood flow in the microfluidic channel. The proposed method can detect the contribution of RBC sedimentation to blood pressure and viscosity. Thus, it can be used to determine whether the blood hematocrit remains constant in the channel during blood delivery with a syringe pump.

## Figures and Tables

**Table 1:** Numerical value of each variable for calculating *X_esr_* and ESR.

Variables	Value	Unit
*t*_0_	881	s
*t*_1_	265	s
*Q_sp_*	5.0	mL/h
*A_ds_*	17.9 × 10^−6^	m^2^
*X_esr_*	20.51	mm
ESR	83.81	mm/h

## Data Availability

The original contributions presented in this study are included in the article. Further inquiries can be directed to the corresponding author.
